# RNA editing of the *AMD1* gene is important for ascus maturation and ascospore discharge in *Fusarium graminearum*

**DOI:** 10.1038/s41598-017-04960-7

**Published:** 2017-07-04

**Authors:** Shulin Cao, Yi He, Chaofeng Hao, Yan Xu, Hongchang Zhang, Chenfang Wang, Huiquan Liu, Jin-Rong Xu

**Affiliations:** 1 State Key Laboratory of Crop Stress Biology for Arid Areas, Purdue-NWAFU Joint Research Center, College of Plant Protection, Northwest A&F University, Yangling, Shaanxi 712100 China; 2 College of Life Sciences, Northwest A&F University, Yangling, China; 3 Department of Botany and Plant Pathology, Purdue University, West Lafayette, IN 47907 USA

## Abstract

Ascospores are the primary inoculum in the wheat scab fungus *Fusarium graminearum* that was recently shown to have sexual stage-specific A-to-I RNA editing. One of the genes with premature-stop-codons requiring A-to-I editing to encode full-length functional proteins is *AMD1* that encodes a protein with a major facilitator superfamily (MFS) domain. Here, we characterized the functions of *AMD1* and its UAG to UGG editing event. The *amd1* deletion mutant was normal in growth and conidiation but defective in ascospore discharge due to the premature breakdown of its ascus wall in older perithecia, which is consistent with the specific expression of *AMD1* at later stages of sexual development. Expression of the wild-type or edited allele of *AMD1* but not un-editable allele rescued the defects of *amd1* in ascospore discharge. Furthermore, Amd1-GFP localized to the ascus membrane and Amd1 orthologs are only present in ascocarp-forming fungi that physically discharge ascospores. Interestingly, deletion of *AMD1* results in the up-regulation of a number of genes related to transporter activity and membrane functions. Overall, these results indicated that Amd1 may play a critical role in maintaining ascus wall integrity during ascus maturation, and A-to-I editing of its transcripts is important for ascospore discharge in *F*. *graminearum*.

## Introduction

*Fusarium graminearum* is one of the causal agents of Fusarium Head Blight (FHB) or scab, a destructive disease of wheat and barley worldwide. Besides causing severe yield losses, the pathogen often contaminates infested grains with deoxynivalenol (DON), zearalenone, and other mycotoxins^1, 2^. *F*. *graminearum* overwinters on plant debris and discharges ascospores into the air in the spring to infect flowering wheat or barley heads. Unlike many other plant pathogenic fungi, sexual reproduction plays a critical role in the infection cycle of *F*. *graminearum* because ascospores are the primary inoculum of FHB^3, 4^. Under field conditions, conidia produced on diseased plant tissues are mainly for spreading infection to vegetative tissues of host plants because of the flowering time of wheat heads.

*F*. *graminearum* is a homothallic ascomycete and a tractable genetic system for studying sexual development because of its high homologous recombination frequency and fertility^5–7^. In the past decade, numerous genes important for sexual reproduction have been identified, including a number of protein kinase, phosphatase, and transcription factor genes and other genes with diverse functions^7–10^. Whereas many of these genes also are important for vegetative growth and asexual reproduction, some have specific functions during sexual reproduction in *F*. *graminearum*, such *GEA1* and *PUK1* that have no other defects but ascospore release or morphology^11, 12^. Interestingly, for the two paralogs of CDK kinase Cdc2 and beta-tubulin, whereas they have overlapping function in vegetative growth, only Cdc2A and Tub1 are important for ascus and ascospore development^13, 14^, suggesting differences in cell cycle regulation and microtubule cytoskeleton between vegetative hyphae and ascogenous tissues in *F*. *graminearum*.

Recently, A-to-I RNA editing was found to specifically occur during sexual reproduction in *F*. *graminearum* ^12^. In animals, A-to-I editing catalyzed by the adenosine deaminase acting on RNA (ADAR) enzymes is the most prevalent type of RNA editing^15^. Although plants and fungi lack ADAR orthologs, more than 26,000 A-to-I editing sites were identified in *F*. *graminearum*, and majority of them occurred in the coding regions and caused amino acid changes^12^. The *PUK1* protein kinase gene known to be important for ascospore development and release^8^ had two tandem premature stop codons UAG UAG in its open reading frame (ORF) that were edited to UGG UGG during sexual reproduction to encode full-length proteins^12^. Additional 69 genes with premature stop codons in their ORFs that had *PUK1*-like editing events in perithecia were identified in *F*. *graminearum* ^12^, suggesting the importance of RNA editing during sexual reproduction.

FGRRES_10094 (=FGSG_10094 of the previous annotation by the Broad Institute) was one of the five hypothetical genes with *PUK1*-like editing events that were selected for preliminary analysis for their roles in sexual reproduction^12^. In this study, we further characterized the functions of FGRRES_10094 (named *AMD1* for ascus maturation and ascospore discharge 1) and its UAG to UGG editing event in ascospore development and release. The *amd1* deletion mutant was defective in ascospore discharge, likely due to the premature breakdown of its ascus wall. In addition to stage-specific editing, *AMD1* was specifically expressed at late stages of sexual development and its orthologs are only present in ascocarp-forming fungi. Expression of different mutant alleles of *AMD1* confirmed the importance of RNA editing. Furthermore, Amd1-GFP localized to the ascus membrane and deletion of *AMD1* results in the up-regulation of a number of genes related to transporter activity and membrane functions. Overall, our results indicated that *AMD1* may play a critical role in maintaining ascus wall integrity and A-to-I editing of its transcripts is important for ascospore discharge and auto-inhibition of ascospore germination in *F*. *graminearum*.

## Results

### *AMD1* encodes a protein unique to ascocarp-forming ascomycetes

**Figure 1:**
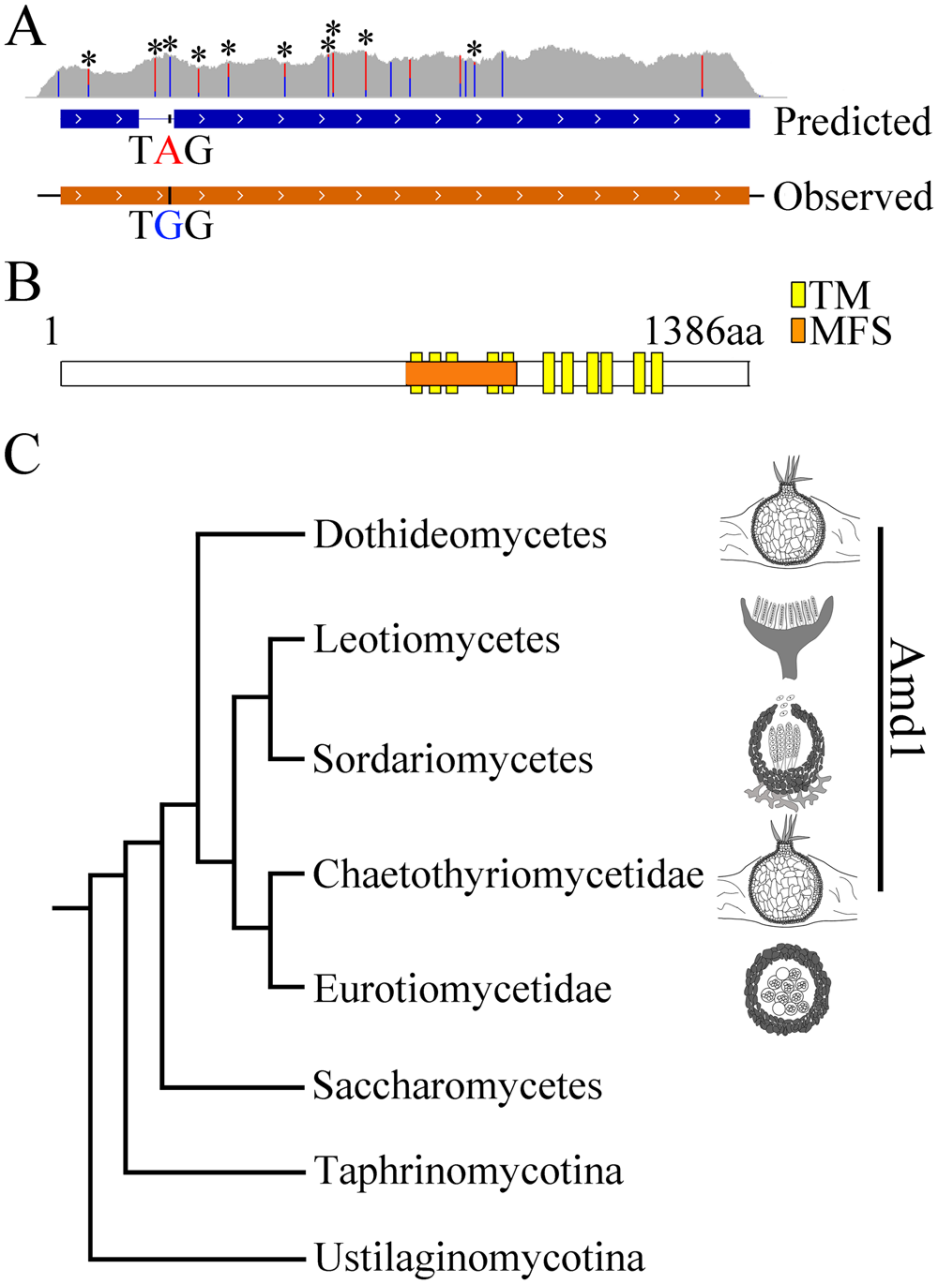
Editing sites and domain structures of *AMD1* and its phylogenetic distribution. **(A)** Transcripts in RNA-seq data, the predicted gene model, and observed coding region of *AMD1*. The actual gene model contains one stop codon UA^632^G in the incorrectly predicted intron that was edited to UG^632^G. Reads coverage from RNA-seq data was in gray shade. Blue and red vertical lines represent the edited and unedited portions of *AMD1* transcripts at each editing site, with nonsynonymous editing sites marked with asterisks. (**B**) The Amd1 protein contains one major facilitator superfamily (MFS) domain (aa. 618–840) and 11 transmembrane helixes (TM). (**C**) The distribution of Amd1 orthologs is restricted to Sordariomycetes (perithecium), Dothidiomycetes (pseudothecium or ascostrama), Leotiomycetes (apothecia), Chaotothriomycetidae (ascostroma) and Eurotiomycetidae (cleistothecia) species of Eurotiomycetes.

The ORF of FGRRES_10094 (named *AMD1* for ascus maturation and ascospore discharge 1) was predicted to contain one intron towards its 5’-end. However, our RNA-seq data^12^ showed that this intron was incorrectly predicted but the stop codon UAG (631–633) within it was changed to UGG by RNA editing in 97.6% of the *AMD1* transcripts in perithecia harvested at 8 days post-fertilization (dpf) (Fig. 1A). The actual *AMD1* ORF encodes a 1386 amino acid protein that contains a well-conserved major facilitator superfamily (MFS) domain and 11 transmembrane helixes (TM) (Fig. 1B). Interestingly, Amd1 appears to be a protein unique to ascocarp-forming ascomycetes because it lacks a distinct ortholog in the budding and fission yeasts and other Taphrinomycotina and Saccharomycotina species (Fig. 1C; Fig. S1). Amd1 orthologs are well conserved in Sordariomycetes, Dothideomycetes, and Leotiomycetes but not in Eurotiomycetes except Chaetothyriomycetidae species (Fig. 1C; Fig. S1). The distribution of Amd1 orthologs suggests that it may be functionally related to physical discharge of ascospores from asci and ascocarps (Fig. 1C).

### The expression of *AMD1* is specific to late stages of sexual development

**Figure 2:**
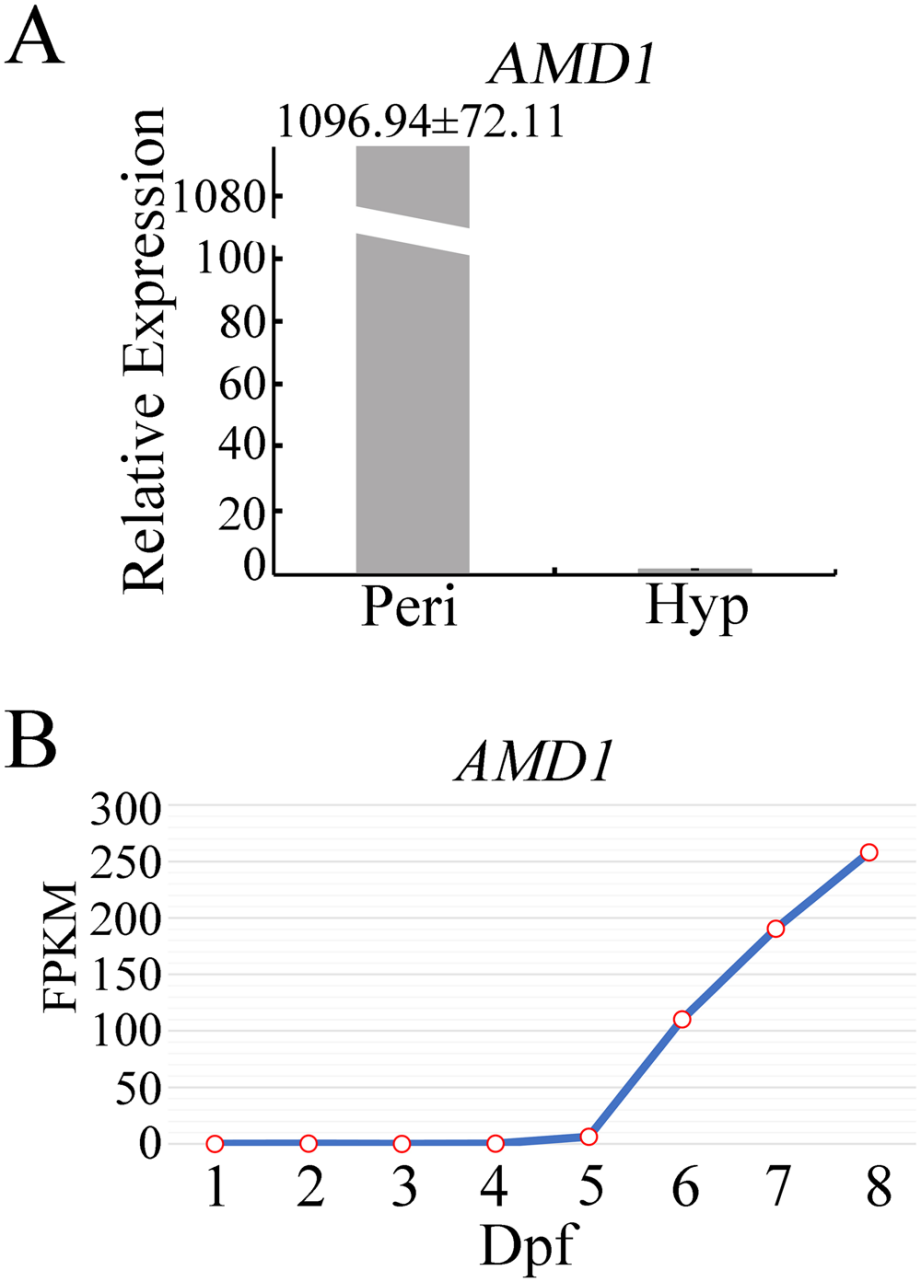
Expression of *AMD1* in late stages of sexual development. **(A)** The expression level of *AMD1* transcripts was assayed by qRT-PCR with RNA isolated from 12 h YEPD cultures (Hyp; arbitrarily set to 1) and 8 days post-fertilization (dpf) perithecia (Peri). Mean and standard deviation were calculated with data from three independent replicates. (**B**) The abundance of *AMD1* transcripts in different sexual stages based on RNA-seq data of mating cultures collected at 1–2 dpf and perithecia sampled at 3–8 dpf. FPKM: Fragments Per Kilobase of exon per Million fragments mapped.

Unlike in perithecia, *AMD1* transcripts were rare in RNA-seq data of hyphae and conidia^12^, suggesting that *AMD1* was almost specifically expressed in perithecia. To verify this result, we assayed *AMD1* expression in PH-1 by qRT-PCR with RNA isolated from 12 h YEPD cultures and 8 dpf perithecia. Consistent with RNA-seq data, *AMD1* transcription was barely detectable in vegetative hyphae but its expression increased over 1,000 folds in perithecia (Fig. 2A).

In RNA-seq generated with RNA isolated from mating cultures sampled at 1 and 2 dpf and perithecia collected 3–8 dpf (accession no. PRJNA384311), *AMD1* expression was barely detectable at early stages but began to increase at 5 dpf (Fig. 2B). The abundance of *AMD1* transcripts kept increasing from 6, 7, and 8 dpf (Fig. 2B). In comparison with 3 dpf young perithecia, the expression level of *AMD1* was up-regulated over 250 folds at 8 dpf. The timing of un-regulated expression of *AMD1* correlates with the ascus and ascospore development in perithecia.

### Ascospore discharge is blocked in the *amd1* mutant

The *AMD1* gene replacement construct was generated and transformed into the wild-type strain PH-1 in a previous study^12^. The *amd1* mutant (Table 1) was normal in vegetative growth and conidiation. In comparison with PH-1, it had no obvious defects in virulence in infection assays with corn silks and wheat heads (Fig. S2). The *amd1* mutant also was normal in response to various stresses, including treatments with 0.75% SDS, 0.05% H_2_O_2_, and 0.7 M NaCl (Fig. S3). These results indicated that, consistent with stage-specific expression during sexual reproduction, *AMD1* is not important for hyphae growth, asexual reproduction, virulence, and stress response.

**Table 1:** The wild-type and mutant strains of *Fusarium graminearum* used in this study.

Strains	Brief descriptions	Reference
PH-1	Wild-type	28
K3	*Fgkin1* deletion mutant of PH-1	8
M3	*amd1* deletion mutant of PH-1	12
M18	*amd1* deletion mutant of PH-1	12
M19	*amd1* deletion mutant of PH-1	12
M22	*amd1* deletion mutant of PH-1	12
NC7	*amd1*/*AMD1* ^WT^-GFP transformant	This study
NE9	*amd1*/*AMD1* ^TGG^ *-GFP* transformant	This study
NT6	*amd1*/*AMD1* ^TAA^ *-GFP* transformant	This study
RE11	*amd1*/P_RP27_-*AMD1* ^TGG^-GFP transformant	This study

**Figure 3:**
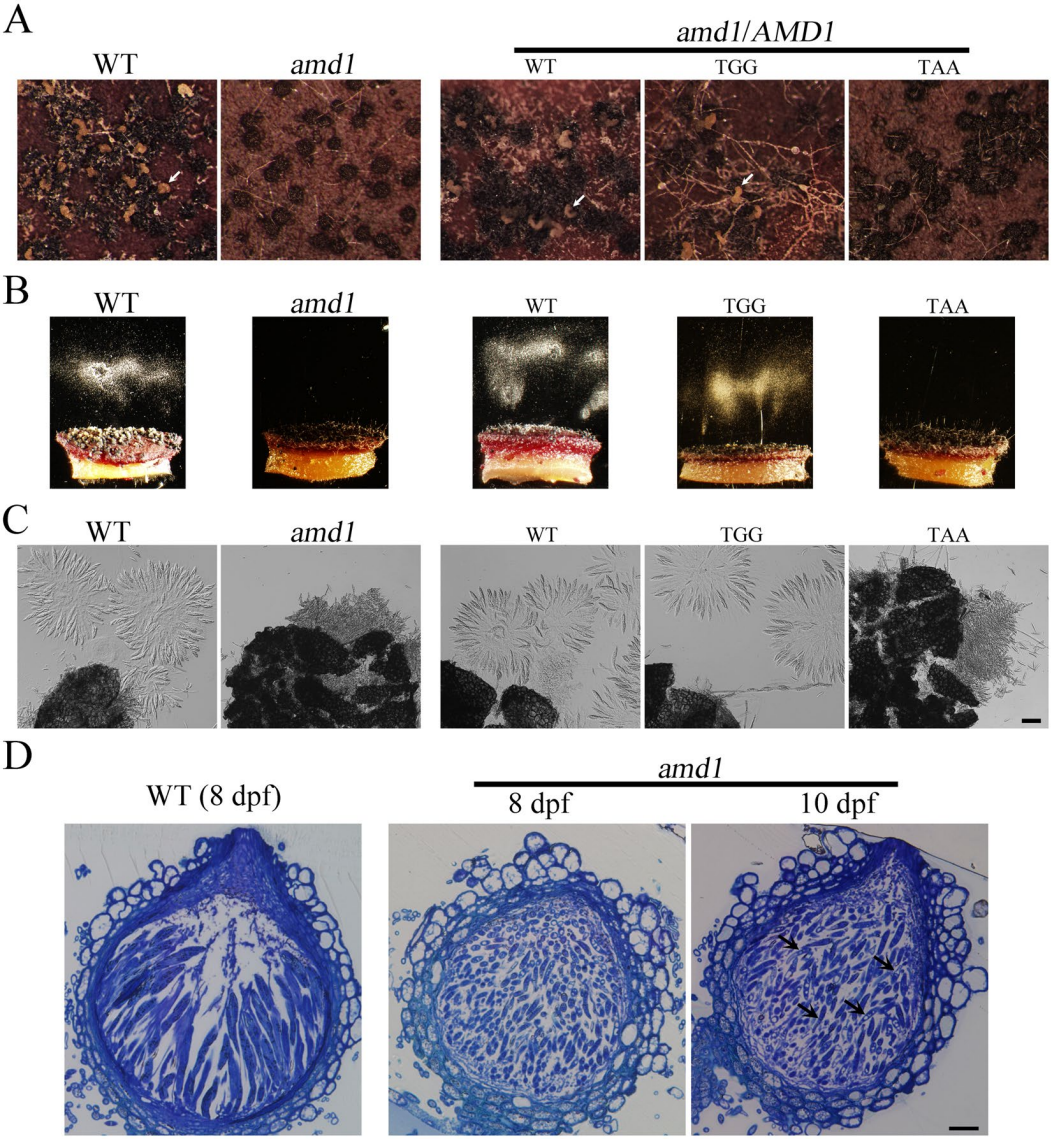
The *amd1* mutant was defective in ascospore release and ascus wall integrity. **(A)** Mating cultures of the wild-type PH-1 (WT), *amd1* mutant, and transformants of *amd1* expressing the *AMD1* ^WT^-, *AMD1* ^TGG^-, or *AMD1* ^TAA^-GFP construct were examined 8 days post-fertilization (dpf). Arrows point to cirrhi. (**B)** Ascospore discharge was assayed with 7 dpf perithecia of the same set of strains. Ascospores discharged from perithecia were accumulated as whitish masses when examined after incubation for 16 h. (**C)** The same set of strains were examined for asci and ascospores in 8 dpf perithecia. No intact asci were observed in the *amd1/AMD1* ^TAA^ transformant. Bar = 20 μm. (**D)** Semi-thin sections of representative perithecia of PH-1 (WT) and the *amd1* mutant that were fixed and stained with 0.5% (wt/vol) toluidine blue. Arrows mark the germinated ascospores. Bar = 20 μm.

The *amd1* mutant also was normal in perithecium development and formed abundant melanized perithecia on carrot agar cultures at 7 dpf. However, ascospore cirrhi were rarely observed in mutant perithecia (Fig. 3A) even after prolonged incubation, suggesting its defects in ascospore release. To confirm this observation, we assayed ascospore discharge as previously described^16^. Whereas abundant ascospores were forcibly discharged from wild-type perithecia after incubation for 16 h, under the same conditions, ascospore discharge was not observed in the *amd1* mutant (Fig. 3B). Therefore, *AMD1* is essential for forcible discharge of ascospores from perithecia in *F*. *graminearum*.

### *AMD1* is required for ascus wall integrity

Although the *amd1* mutant was defective in ascospore discharge, they formed abundant ascospores inside perithecia. However, when 8 dpf perithecia were examined, only scattered ascospores but not intact asci were observed in the *amd1* mutant (Fig. 3C), suggesting the breakdown of ascus wall. Under the same conditions, fascicles of asci were present in wild-type perithecia (Fig. 3C). To verify this observation, we examined perithecia with semi-thin sections of 8 dpf perithecia. In the wild type, asci with ascospores were observed (Fig. 3D). However, only scattered ascospores but not asci were observed in mutant perithecia (Fig. 3D). Because turgor pressure inside asci is important for the forcible discharge of ascospores, the premature breakdown of ascus wall in mutant perithecia may be directly responsible for its defects in ascospore release and formation of ascospore cirrhi.

**Figure 4:**
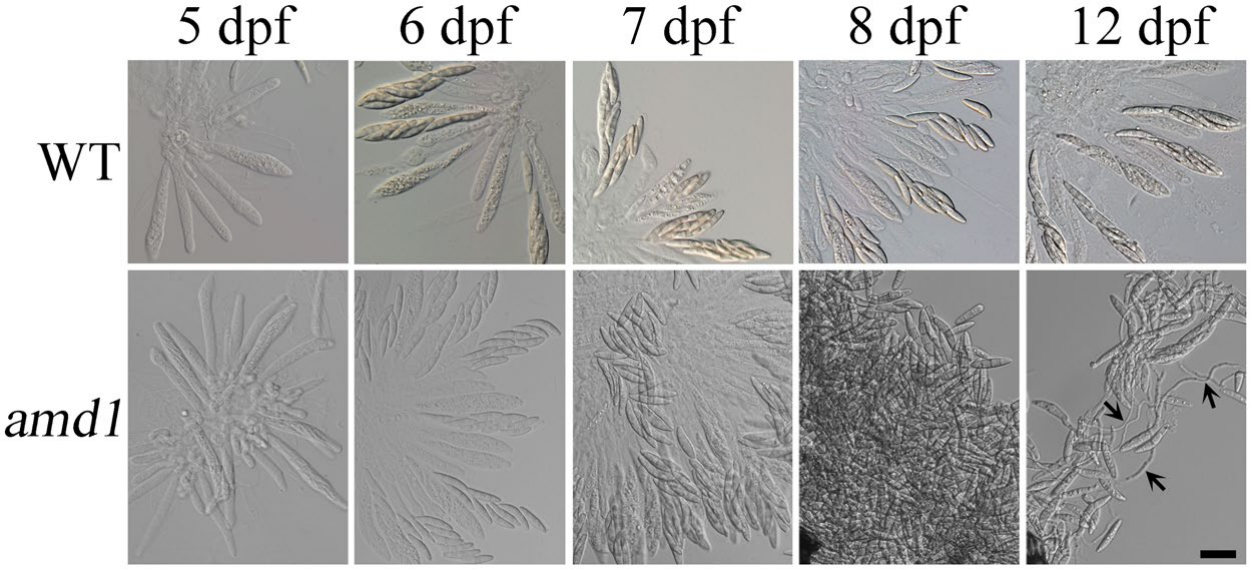
Ascus and ascospore development in the wild type and *amd1* mutant. Perithecia of the wild-type strain PH-1 (WT) and the *amd1* mutant were examined at 5, 6, 7, 8, and 12 days post fertilization (dpf). Bar = 20 μm. Arrows mark the germinated ascospores in the mutant.

To determine the timing of ascus wall disintegration, we examined ascospores and asci in perithecia sampled at 5, 6, 7, and 8 dpf. Both the wild type and *amd1* mutant strains had fascicles of asci with 8-ascospores in 5 or 6 dpf perithecia. In 7 dpf perithecia, the ascus wall begun to disintegrate and the arrangement of ascospores in asci became loose in the *amd1* mutant (Fig. 4). No asci were observed in mutant perithecia at 8 dpf (Fig. 4). These results indicate that the breakdown of ascus wall began at 7 dpf and completed by 8 dpf.

### Germination of *amd1* ascospores inside perithecia

Similar to the wild type, the *amd1* mutant still produced four-celled ascospores. However, most of the mutant ascospores had germinated inside perithecia by 12 dpf (Fig. 4). Ascospore germination also was visible in semi-thin sections of mutant perithecia sampled at 10 dpf (Fig. 3D). Under the same conditions, ascospore germination was never observed inside perithecia formed by the wild type (Fig. 4). Extensive observations with mutant perithecia showed that ascospore germination only occurred after the breakdown of the ascus wall. Germination was not observed with ascospores that were still inside intact asci. These results showed that ascus wall integrity is important for preventing ascospore germination inside perithecia in *F*. *graminearum*.

**Figure 5:**
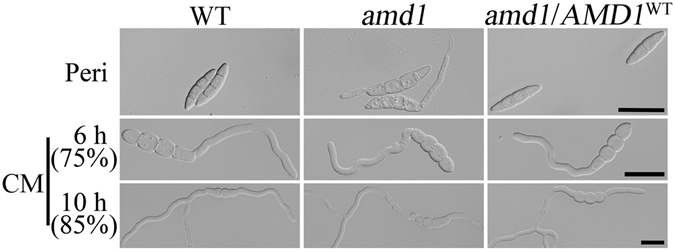
Germination of *amd1* mutant ascospores in perithecia and CM cultures. Ascospores of the wild-type strain PH-1 (WT), *amd1* mutant, and *amd1/AMD1* ^WT^-GFP complemented transformant *amd1/AMD1* ^WT^ were examined for germination in 12 dpf perithecia (Peri) or after incubation in complete medium (CM) at 25 °C for 6 h and 10 h. Ascospore germination was not observed in the wild type but germination from one end of ascospores was observed in the *amd1* mutant. When incubated in CM, germination from both ends of ascospores increased from approximately 25% at 6 h to 85% at 10 h in both the wild type and *amd1* mutant. Bar = 20 μm.

Interestingly, germ tubes were produced only from one end of mutant ascospores when they germinated inside perithecia (Fig. 5). When incubated in liquid complete medium (CM), both the wild-type and mutant ascospores first produced germ tubes from one end but germination from the other end also occurred rapidly. After incubation in CM for 6 h CM, approximately 25% of ascospores had germ tubes from both ends. The percentage of ascospores germinated from both ends increased to 85% in 10 h CM cultures (Fig. 5). These results indicated that mutant ascospores germinated in different manners under different conditions. Unlike germination in nutrient media, germination inside perithecia may involve different regulatory mechanisms.

### The *AMD1*^WT^ and *AMD1*^TGG^ but not *AMD1*^TAA^ alleles complement the *amd1* mutant

For complementation assays, the wild-type *AMD1* allele with the TA^632^G stop codon and its promoter region was amplified and fused with GFP to generate the *AMD1* ^WT^-GFP construct, which was then transformed into the *amd1* mutant. All the resulting *amd1*/*AMD1* ^WT^-GFP transformants were normal in hyphal growth, conidiation, and sexual reproduction. Perithecia formed by the *amd1*/*AMD1* ^WT^-GFP transformants formed ascospore cirrhi and had no ascospore germination inside perithecia (Fig. 3A), indicating the complementation of *amd1*.

To determine the function of A^632^-to-I editing, we also generated the *AMD1* ^TGG^-GFP (edited) and *AMD1* ^TAA^ (uneditable) constructs by introducing the A632G and G633A mutations, respectively, and transformed them into the *amd1* mutant. The resulting transformants were screened by PCR and examined for defects in sexual reproduction. All the *amd1*/*AMD1* ^TAA^ transformants had similar defects with the original *amd1* mutant in ascospore release and ascus wall integrity (Fig. 3A), indicating the essential role for RNA editing in *AMD1* function. However, expression of the *AMD1* ^TGG^ allele fully complemented the ascospore release defects of *amd1* (Fig. 3A). The *amd1*/*AMD1* ^TGG^-GFP transformants were normal in ascospore discharge and formed ascospore cirrhi as frequently as the wild type. Therefore, expression of *AMD1* ^TGG^, similar to the wild-type allele, fully complemented the *amd1* mutant, suggesting that the unedited transcripts (2.4%) of *AMD1* had no functions during sexual reproduction.

### Amd1-GFP localizes to the ascus membrane

**Figure 6:**
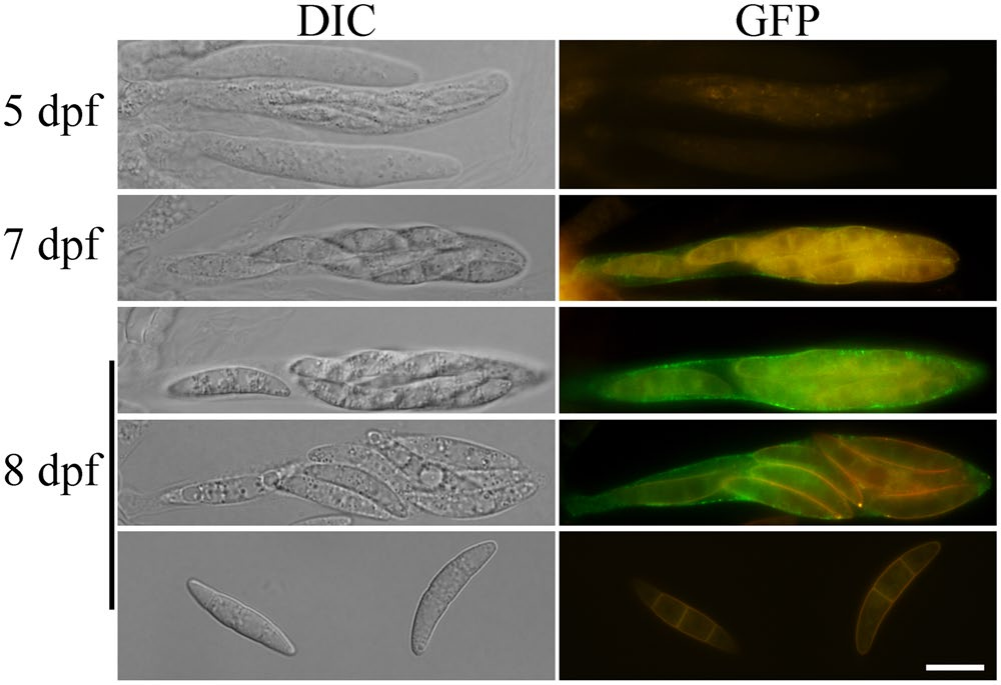
Expression and localization of Amd1-GFP fusion proteins. Asci and ascospores from 5, 7 and 8 dpf perithecia of the *amd1*/*AMD1* ^WT^-GFP transformant were examined by DIC and epifluorescence microscopy. Bar = 10 μm. Ascospores outside asci had no GFP signals.

None of the *amd1*/*AMD1* ^WT^-GFP transformants had detectable GFP signals in vegetative hyphae and conidia (Fig. S4), which was consistent with the specific expression of *AMD1* in perithecia. When perithecia of different development stages were examined, no GFP signals were observed in asci of 5 dpf perithecia or ascospores outside asci in older perithecia (Fig. 6). Amd1-GFP mainly localized to the ascus membrane in 8 dpf perithecia (Fig. 6). To our knowledge, this is the first report on proteins localizes to the ascus membrane in filamentous ascomycetes. The subcellular localization pattern of Amd1 is consistent with its TM helixes and functions in maintaining ascus wall integrity and ascospore discharge in *F*. *graminearum*.

### Constitutive expression of the *AMD1*^TGG^ allele has no effects on hyphal growth and conidiation

Although *AMD1* transcripts were rare in hyphae, it is possible that the existence of the UA^632^G stop codon is to avoid accidental expression of Amd1 proteins, which may be detrimental to vegetative growth in *F*. *graminearum*. To test this hypothesis, we generated the P_RP27_-*AMD1* ^TGG^-GFP construct and transformed it into the *amd1* mutant. The resulting transformants had no obvious defects in vegetative growth and conidiation (Table S1). In 8 h germlings, localization of Amd1-GFP to the cytoplasm membrane was not observed but GFP signals were observed in peri-nuclear regions that may be related to the endoplasmic reticulum due to overexpression (Fig. S4). These results indicate that expression of *AMD1* ^TGG^ by the strong, constitutive RP27 promoter ^17, 18^ had no effects on vegetative growth and asexual reproduction, and the localization of Amd1 to the ascus membrane may depend on its interacting proteins that are specifically expressed during sexual reproduction.

### The expression of *AMD1* is reduced in the *Fgkin1* mutant

**Figure 7:**
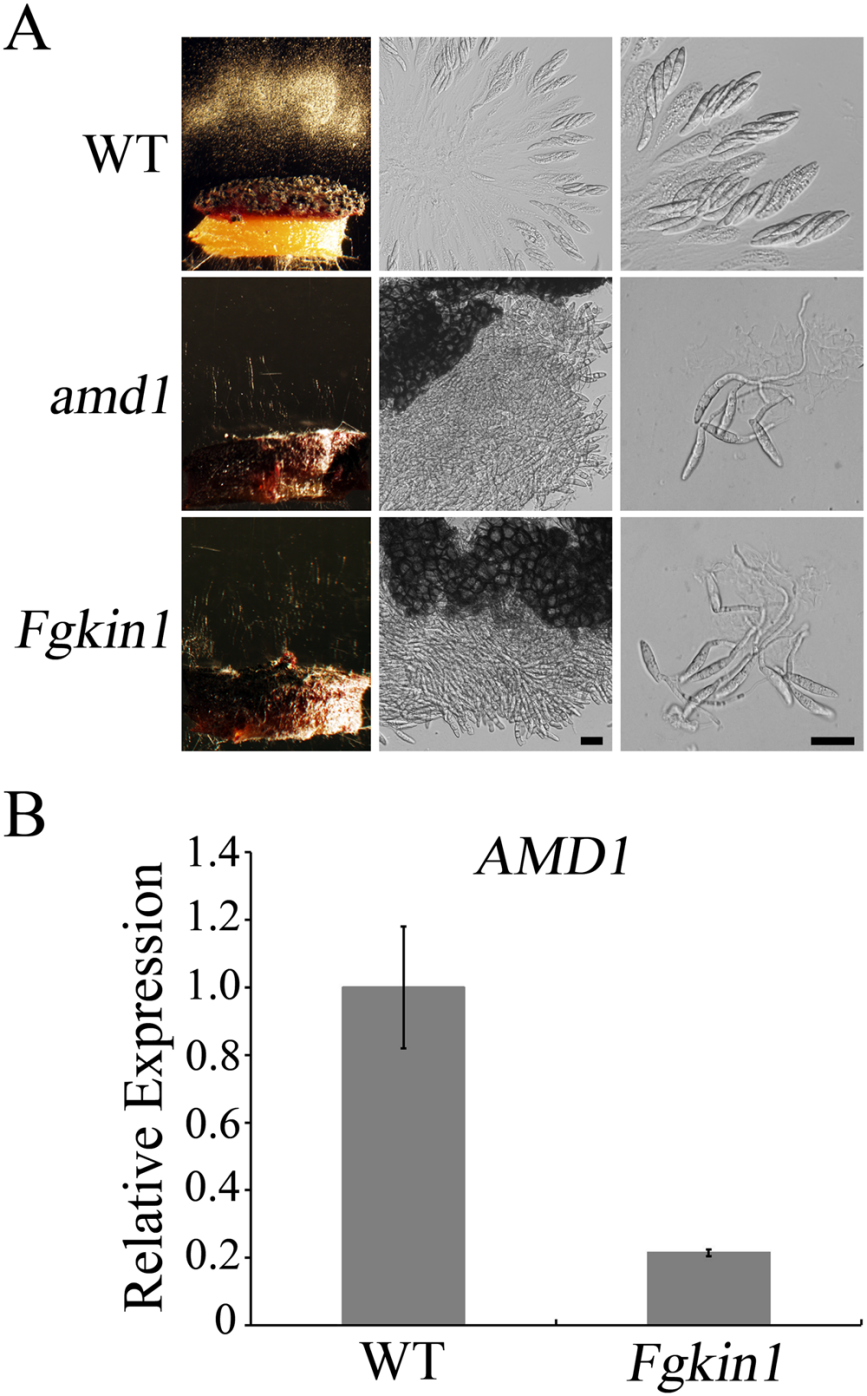
Similarity between *Fgkin1* and *amd1* mutants and reduced *AMD1* expression in *Fgkin1*. **(A)** The *Fgkin1* and *amd1* mutants had similar defects in ascospore discharge, ascus wall disintegration, and ascospore germination inside perithecia. Bar = 20 μm. (**B)** The expression level of *AMD1* was assayed by qRT-PCR with RNA isolated from 7 dpf perithecia of the wild-type PH-1 and *Fgkin1* mutant. The expression level of *AMD1* in PH-1 was arbitrarily set to 1. Mean and standard deviation were calculated with data from three independent replicates.

In *F*. *graminearum*, FgKin1, a microtubule affinity-regulating protein kinase (MARK), is also required for ascospore discharge and prevention of ascospore germination inside perithecia^19^. The *Fgkin1* and *amd1* mutants has similar defects in ascospore discharge and disintegration of the ascus wall (Fig. 7A). Similar to *amd1*, germination of ascospores from one end also was observed inside *Fgkin1* perithecia^19^ (Fig. 7A). When assayed by qRT-PCR with RNA isolated from 7 dpf perithecia, the *AMD1* expression level was reduced approximately 5 folds in the *Fgkin1* mutant in comparison with that of the wild type (Fig. 7B). It is possible that the FgKin1 kinase controls ascus wall integrity by somehow regulating the expression of *AMD1* in *F*. *graminearum*.

### Deletion of *AMD1* affects more than 300 genes expression

To identify genes affected by *AMD1* deletion, we conducted RNA-seq analysis with RNA isolated from perithecia sampled at 7 dpf. In comparison with the wild type, 53 and 263 genes were up- and down-regulated over two folds, respectively, in the *amd1* mutant (Table S2). Among the up-regulated genes, Gene Ontology (GO) enrichment analysis showed that 19 genes each related to transporter activity and membrane were significantly enriched (Fig. S5), suggesting that deletion of *AMD1* may affect cross-membrane transportation and membrane functions. Among the down-regulated genes, approximately half of them encode hypothetical proteins or proteins of unknown functions (Table S2) and no significant enrichment of any GO terms was observed. However, several genes that may be related to cell wall synthesis, modifications, or integrity were down-regulated in the mutant, including FGRRES_12586, FGRRES_07238, FGRRES_17404, FGRRES_02262, FGRRES_10920, FGRRES_13169, and FGRRES_03674. Reduced expression of these genes may be related to the defects of *amd1* in ascus wall integrity.

## Discussion

The *AMD1* gene requires A-to-I RNA editing during sexual reproduction to encode a full-length protein. Interestingly, its orthologs are only present in ascomycetes that form asci inside ascocarps and eject ascospores from asci. Most of the Eurotiomycetes that form cleistothecia such as *Aspergillus* *nidulans* lack *AMD1* orthologs. It is tempting to speculate that *AMD1* is functionally related to the physical ejection of ascospores from asci and its orthologs may evolve only in ascomycetes that are more advanced than those forming cleistothecia^20^. Ascospores are not ejected from naked asci formed by Taphrinomycotina and Saccharomycotina species or cleistothecia formed by Eurotiomycetes. In fact, Chaetothyriomycetidae species that have *AMD1* orthologs are distinct from the rest of Eurotiomycetes by the formation of ascostroma and many of them are lichen forming fungi^21^.

The forcible ejection of ascospores is functionally related to the generation of turgor pressure in asci^22, 23^. In *F*. *graminearum*, individual mature asci extend through the ostiole prior to ascospore discharge^24^. It is likely that the ascus wall was degraded in the *amd1* mutant before asci were mature and ready for ascospore ejection. Although its exact function is not clear, *AMD1* may be involved in strengthening or the modification of ascus wall at later stages. It is also possible that mannitol accumulation and ion fluxes important for ascus turgor generation^23^ were affected in the *amd1* mutant, which in turn may affect ascus turgor and ascus wall modifications. The specific expression of *AMD1* at late sexual stages and its localization to the ascus membrane supported the likely functions of Amd1 proteins in maintaining ascus wall integrity. Furthermore, RNA-seq analysis showed that several genes related to cell wall modification or integrity were down-regulated in mutant perithecia. Interestingly, 19 genes each encoding proteins that are functionally related to transporter activity and membrane functions were upregulated in the *amd1* mutant, which accounted for over two thirds of the 53 up-regulated genes. Most of these genes with up-regulated expression in *amd1* had no or little expression in 8 dpf perithecia in the wild type^12^, suggesting that their up-regulation may be related to the breakdown of ascus wall and membrane in the mutant.

Another defect of the *amd1* mutant was the germination of ascospores from one end inside perithecia after the breakdown of ascus wall. This defect is similar to that of the *Fgkin1* mutant^19^. Kin1 is a MARK kinase that is involved in microtubule based transportation via phosphorylation of microtubule-associated proteins^25^. In this study, we showed that the expression level of *AMD1* was decreased approximately 5 folds in the *Fgkin1* mutant, which may be directly related to the defects of *Fgkin1* in ascospore discharge and germination. Because Amd1 localized to the ascus membrane but FgKin1 localizes to the septal pore^19^, they may not directly interact with each other and *AMD1* expression may be indirectly regulated by *FgKIN1*. Nevertheless, unlike *AMD1*, *FgKIN1* is constitutively expressed, and the *Fgkin1* mutant had a reduced growth rate^19^. Therefore, the FgKin1 kinase must have other downstream targets and more diverse functions than Amd1 in *F*. *graminearum*. The *gea1* mutant is another mutant in *F*. *graminearum* that had similar defects with *amd1* in ascospore discharge and germinati^11^. However, different from Amd1, Gea1 protein localizes to the cytoplasm membrane of ascospores and some *gea1* ascospores had morphology defects. Nevertheless, it will be important to determine the relationships among Amd1, Fgkin1, and Gea1 during ascus maturation and ascospore ejection.

Like the *Fgkin1* mutant^19^, ascospores of the *amd1* mutant germinated from one end inside perithecia but germinated from both ends when cultured in CM. These observations suggest that the two ends of mature ascospores are not equal and the presence of nutrients may promote the production of germ tubes from both ends of ascospores in *F*. *graminearum*. However, it is puzzling how the fungus distinguishes one end from the other in four-celled ascospores derived from two rounds of mitosis and cytokinesis. It is also not clear what molecular mechanisms are responsible for the auto-inhibition of ascospore germination inside perithecia. If *F*. *graminearum* produces certain metabolites or ascospore surface compounds that function as auto-inhibitory factors to prevent ascospores from germination inside perithecia, the *amd1* and *Fgkin1* mutants may be defective in the production or accumulation of these compounds. It will be important to assay for defects of the *amd1* and *Fgkin1* mutants in the accumulation of mannitol and ions enriched inside asci^4, 26, 27^ if they are responsible for auto-inhibition of ascospore germination in *F*. *graminearum*.

*AMD1* is one of the 60 genes with premature stop codons in the coding regions that require A-to-I editing to encode full-length proteins in *F*. *graminearum* ^12^. Because RNA editing is incomplete, even though the editing level was 97.6% at A632, the unedited transcripts were still present and might encode a small peptide. Nevertheless, the *amd1*/*AMD1* ^TGG^-GFP transformants were similar to the wild type and complemented transformant in ascospore discharge, indicating that the unedited transcripts had no detectable functions if they indeed encoded a small peptide in *F*. *graminearum*. However, there are 9 other nonsynonymous editing events identified in the *AMD1* transcripts, including two in the MFS domain (Fig. 1A). Five of editing sites have editing levels higher than 90% and another 7 had editing levels ranging from 30–90%. Therefore, RNA editing not only enables *AMD1* to encode a full-length functional protein but also introduces amino acid sequence variations in *F*. *graminearum*. It will be interesting to determine the functions of these nonsynonymous editing sites in *AMD1*.

## Methods

### Strains and culture conditions

The *F*. *graminearum* wild-type strain PH-1^28^ and all the transformants generated in this study were routinely maintained on potato dextrose agar (PDA) plates at 25 °C. Conidiation in liquid carboxymethyl cellulose (CMC) medium and growth rate on complete medium (CM) plates were measured as described^29, 30^. To assay for defects in stress responses, final concentrations of 0.75% sodium dodecyl sulfate (SDS), 0.1% H_2_O_2_, and 0.7 M NaCl were added to CM as described^8^. For sexual reproduction, aerial hyphae of 5-day-old carrot agar cultures were pressed down with sterile 0.1% Tween 20^6, 31^. Perithecia, cirrhi, asci, and ascospore discharge were examined as described^16, 19^. Protoplast preparation and polyethylene glycol (PEG)-mediated transformation were performed as described^29^. Hygromycin B (CalBiochem, La Jolla, CA, USA) and geneticin (Sigma-Aldrich, St. Louis, MO, USA) were added to the final concentration at 300 and 400 μg/ml, respectively, for transformant selection.

### Generation of the *AMD1*^WT^, *AMD1*^TGG^, *AMD1*^TAA^, and P_Rp27_-*AMD1*^TGG^ transformants

For complementation assays, the entire *AMD1* gene including its promoter region was amplified with primers 094-NF and 094-R (Table S3) and co-transformed with *Xho*I-digested pFL2 (carrying the geneticin resistance marker) into yeast strain XK1–25 by the gap repair approach^17, 32^. The *AMD1* ^WT^-GFP fusion construct was rescued from Trp^+^ yeast transformants and confirmed by sequencing analysis. The same yeast gap repair approach was used to generate the *AMD1* ^TGG^-GFP, P_RP27_ *-AMD1* ^TGG^-GFP, and *AMD1* ^TAA^-GFP constructs. To introduce the A632G and G633A mutations, *AMD1* was amplified with primer pairs 094E-F /094E-R and 094S-F /094S-R (Table S3), respectively. All the resulting mutant alleles of *AMD1* were verified by sequencing and transformed into the *amd1* mutant. Transformants of *amd1* expressing the *AMD1* ^WT^-, *AMD1* ^TGG^ *-*, P_RP27_ *-AMD1* ^TGG^ *-*, and *AMD1* ^TAA^ *-*GFP constructs were identified by PCR and examined for GFP signals by epifluorescence microscopy.

### Specimen preparation for semi-thin sections

Perithecia collected from mating cultures at 8 or 10 dpf were fixed with 4% (vol/vol) glutaraldehyde in 0.1 M phosphate buffer (pH 6.8) overnight at 4 °C. Samples were then dehydrated in a series of acetone consisting of 30, 50, 70, 80, 90, and 100% (vol/vol). The dehydrated samples were embedded in Spurr resin as described^33^. Semi-thin sections (1 μm in thickness) were stained with 0.5% (wt/vol) toluidine blue before being examined with an Olympus BX-53 microscope.

### qRT-PCR analysis

For qRT-PCR assays, RNA samples were isolated with the TRIzol reagent (Invitrogen, Carlsbad, CA, USA) from perithecia collected at 7 dpf. The Fermentas First cDNA synthesis kit (Hanover, MD, USA) was used for cDNA synthesis. The *TUB2* beta-tubulin gene was used as the internal control^34^ and the relative expression of each gene was calculated with the 2^−△△Ct^ method. Data from three biological replicates were used to calculate the mean and standard deviation of the expression levels^35^.

### Plant infection assays

For infection assays with flowering wheat heads of cultivar Xiaoyan 22, conidia were harvested from 5-day-old CMC cultures and re-suspended to 2.0 × 10^5^ conidia/ml in sterile distilled water. For each head, the fifth spikelet from the base of the inflorescence was inoculated with 10 μl of conidium suspensions as described^36, 37^. FHB symptoms were examined at 14 day post-infection to estimate the disease index^38, 39^. Corn silks were infected with culture blocks and examined as described^40^.

### RNA-seq analysis

Perithecia of PH-1 and *amd1* mutant were harvested from carrot agar cultures at 7 dpf and used for RNA extraction with TRIzol (Invitrogen, USA). For each strain, RNA was isolated from two biological replicates. RNA-seq libraries were prepared with the NEBNext^®^ Ultra™ Directional RNA Library Prep Kit (NEB, USA) following the instruction provided by the manufacturer and sequenced with Illumina HiSeq 2500 with the paired-end 2 × 150 bp model at the Novogene Bioinformatics Institute (Beijing, China). For each replicate, at least 24 Mb paired-end reads were obtained. The resulting RNA-seq reads were mapped onto the reference genome of *F*. *graminearum* strain PH-1^28, 41^ by HISAT2^42^. The number of reads (count) mapped to each gene were calculated by featureCounts^43^. Differential expression analysis of genes was performed using the edgeRun package^44^ with the exactTest function. Genes with a FDR (false discovery rate) of below 0.05 and |log_2_FC (log_2_ fold change)| of above 1 were regarded as differentially expressed genes.

### Data availability

RNA-seq data were deposited at NCBI SRA database under accession number SRP100650.

## Electronic supplementary material

Supplementary Information
