# Supplementary material for: RNA editing of the AMD1 gene is important for ascus maturation and ascospore discharge in Fusarium graminearum
Source: Sci Rep. 2017 Jul 4;7:4617. doi: 10.1038/s41598-017-04960-7 (PMC5496914; doi:10.1038/s41598-017-04960-7)
Supplement: Supplementary file 1 — Supplementary Information [file 41598_2017_4960_MOESM1_ESM.pdf]

**RNA editing of the *AMD1* gene is important for ascus maturation and ascospore discharge in *Fusarium graminearum***

Shulin Cao[1], Yi He[2], Chaofeng Hao[1], Yan Xu[1], Hongchang Zhang[2], Chenfang Wang[1], Huiquan Liu[1]*, and Jin-Rong Xu[1,3]*

[1] State Key Laboratory of Crop Stress Biology for Arid Areas, Purdue-NWAFU Joint Research Center, College of Plant Protection, Northwest A&F University, Yangling, Shaanxi 712100, China.

[2] College of Life Sciences, Northwest A&F University, Yangling, China

[3] Department of Botany and Plant Pathology, Purdue University, West Lafayette, IN 47907. USA.

*Corresponding authors: Jin-Rong Xu
Tel: 765-496-6918
jinrong@purdue.edu

Huiquan Liu
Tel: +86-29-87081260
liuhuiquan@nwsuaf.edu.cn

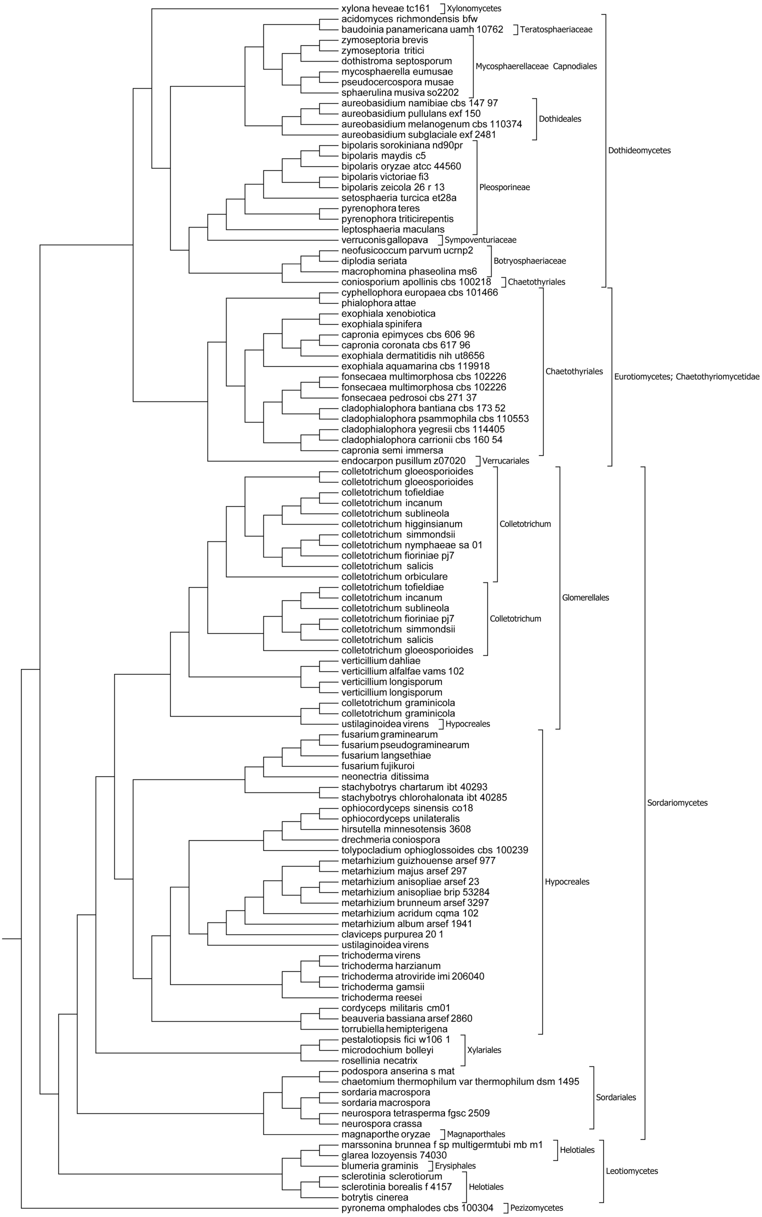


**Fig. S1**. **Phylogenetic distribution of *AMD1* orthologs in ascomycetes.**

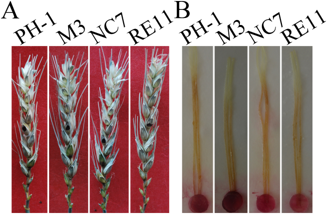


**Fig. S2**. **Infection assays with the *amd1* mutant.** (**A**) Flowering wheat heads were drop-inoculated with conidia from the wild type PH-1 (WT), *amd1* mutant M3, complemented strain NC7, and *amd1*/$P_{RP27}$-*AMD1*$^{TGG}$ transformant RE11 and photographed at 14 days post-inoculation (dpi). Black dots mark the inoculated spikelets. (**B**) Corn silks were inoculated with culture blocks of the same set of strains and photographed at 5 dpi.

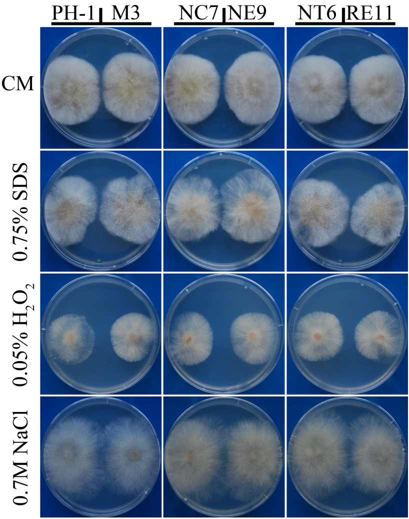


**Fig. S3. Stress response assays with the *amd1* mutant.** Two-day-old cultures of PH-1, *amd1* mutant (M3), and transformants of *amd1* expressing the *AMD1*$^{WT}$ (NC7), *AMD1*$^{TGG}$ (NE9), *AMD1*$^{TAA}$ (NT6), and $P_{RP27}$-*AMD1*$^{TGG}$ (RE11) alleles grown on CM plates with or without 0.05% $H_2O_2$, 0.7 M NaCl, or 0.75% SDS.

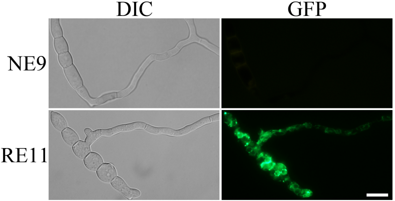


**Fig. S4. Expression of *AMD1* in germlings.** Conidia from transformants of the *amd1* mutant expressing the *AMD1*$^{TGG}$-GFP (NE9) and $P_{RP27}$-*AMD1*$^{TGG}$-GFP (RE11) constructs were incubated in CM for 12 h and examined by DIC and epifluorescence microscopy. Bar = 10 µm.

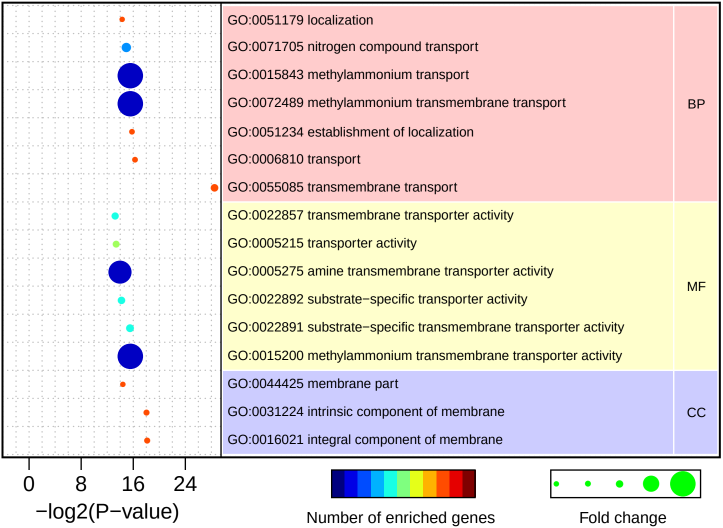


**Fig. S5. Gene ontology (GO) enrichment analysis of the genes upregulated in the *amd1* mutant.** GO terms with adjusted *P*-values (FDR) < 0.05 are presented. BP, MF, and CC stand for biological process, molecular function, and cellular component, respectively.

**Table S1. Assays for vegetative growth, conidiation and pathogenicity in wild type, the *amd1* mutant, *amd1*/*AMD1*$^{WT}$ (NC7), and *amd1*/P$_{RP27}$-*AMD1*$^{TGG}$ transformant RE11.**

| Strain | Growth rate (mm/d)[a] | | Conidiation[b] (×10$^{6}$conidia/ml) | Disease index[c] |
|---|---|---|---|---|
| | PDA | CM | | |
| PH-1 | 12.2±0.1$^{A}$ | 11.2±0.2$^{A}$ | 1.3±0.2$^{A}$ | 10.3±1.2$^{A}$ |
| M3 | 12.1±0.1$^{A}$ | 11.3±0.2$^{A}$ | 1.2±0.1$^{A}$ | 10.1±1.0$^{A}$ |
| NC7 | 12.1±0.1$^{A}$ | 11.2±0.2$^{A}$ | 1.3±0.1$^{A}$ | 10.7±1.7$^{A}$ |
| RE11 | 12.2±0.1$^{A}$ | 11.4±0.2$^{A}$ | 1.2±0.1$^{A}$ | 10.2±1.5$^{A}$ |

Data from three replicates were analyzed with the protected Fisher's Least Significant Difference (LSD) test. The same letter indicated that there was no significant difference ($P \leq 0.05$).

[a] Average growth rate and standard deviation were calculated from at least three independent measurements.

[b] Conidiation in 5-day-old CMC cultures.

[c] Disease was rated by the number of symptomatic spikeletes 14dpi. Mean and standard deviation were calculated with results from three independent replicates. At least 10 wheat heads were examined in each replicate.

**Table S2. Genes differentially expressed at least two-fold in amd1 mutant.**

| Gene ID | logFC* | P-Value | FDR[#] | blastp annotation |
|---|---|---|---|---|
| FGRRES_08104 | 2.52 | 2.13E-16 | 6.03E-14 | hypothetical protein |
| FGRRES_10587 | 2.41 | 2.36E-04 | 5.58E-03 | membrane primary amine oxidase |
| FGRRES_00119 | 2.32 | 6.95E-04 | 1.43E-02 | hypothetical protein |
| FGRRES_20380 | 2.31 | 4.23E-04 | 9.18E-03 | het domain-containing protein |
| FGRRES_09072 | 2.16 | 3.55E-10 | 3.36E-08 | hypothetical protein |
| FGRRES_12213_M | 2.16 | 1.84E-05 | 5.99E-04 | hypothetical protein |
| FGRRES_02038 | 2.09 | 1.35E-05 | 4.55E-04 | quinone oxidoreductase 2 |
| FGRRES_01661 | 1.96 | 1.43E-07 | 7.86E-06 | taurine dioxygenase |
| FGRRES_07790 | 1.91 | 2.30E-05 | 7.29E-04 | allergen fus c 3 |
| FGRRES_03108 | 1.90 | 2.75E-03 | 4.45E-02 | rna exonuclease 3 |
| FGRRES_16063 | 1.85 | 7.52E-06 | 2.72E-04 | cysteine transporter |
| FGRRES_08197_M | 1.84 | 9.15E-07 | 4.14E-05 | aael017480- partial |
| FGRRES_00055 | 1.76 | 5.35E-05 | 1.54E-03 | hypothetical protein |
| FGRRES_03111 | 1.73 | 1.19E-15 | 2.94E-13 | urea active transporter |
| FGRRES_04785 | 1.70 | 5.59E-05 | 1.59E-03 | trichothecene c-8 hydroxylase |
| FGRRES_09104_M | 1.68 | 2.60E-04 | 6.03E-03 | pantothenate transporter liz1 |
| FGRRES_01662 | 1.64 | 6.51E-06 | 2.41E-04 | major facilitator superfamily transporter |
| FGRRES_05681 | 1.60 | 2.63E-08 | 1.68E-06 | ethanolamine utilization protein |
| FGRRES_03898 | 1.59 | 2.04E-12 | 2.75E-10 | fad-binding protein |
| FGRRES_08341 | 1.56 | 2.28E-05 | 7.24E-04 | nicotinamide mononucleotide permease |
| FGRRES_12355 | 1.51 | 1.23E-03 | 2.27E-02 | quinate repressor protein |
| FGRRES_12415_M | 1.51 | 3.15E-03 | 4.96E-02 | tartrate transporter |
| FGRRES_11157 | 1.48 | 8.39E-04 | 1.66E-02 | caib baif family enzyme |
| FGRRES_04848 | 1.45 | 8.54E-04 | 1.68E-02 | rhamnogalacturonan acetylesterase |
| FGRRES_11475 | 1.37 | 1.14E-04 | 3.02E-03 | major facilitator superfamily transporter |
| FGRRES_06513 | 1.36 | 1.95E-05 | 6.30E-04 | high-affinity nickel-transporter |
| FGRRES_17610 | 1.34 | 7.23E-09 | 5.31E-07 | ammonium permease |
| FGRRES_03842 | 1.32 | 5.15E-05 | 1.49E-03 | alpha-amylase |
| FGRRES_07590 | 1.27 | 3.28E-04 | 7.37E-03 | taurine dioxygenase |
| FGRRES_05011 | 1.26 | 2.32E-05 | 7.33E-04 | tartrate transporter |
| FGRRES_16924 | 1.24 | 5.18E-06 | 1.97E-04 | alternative sulfate transporter |
| FGRRES_11433 | 1.24 | 7.55E-04 | 1.52E-02 | hypothetical protein |
| FGRRES_11069 | 1.22 | 2.75E-03 | 4.45E-02 | allantoate transport protein |

| Gene ID | logFC* | P-Value | FDR[#] | blastp annotation |
|---|---|---|---|---|
| FGRRES_03872_M | 1.20 | 4.93E-07 | 2.36E-05 | tartrate transporter |
| FGRRES_09646 | 1.20 | 2.00E-11 | 2.27E-09 | laccase precursor |
| FGRRES_04544_5_M | 1.18 | 8.54E-08 | 4.95E-06 | maltose permease mal31 |
| FGRRES_08048 | 1.17 | 2.51E-03 | 4.12E-02 | regulatory p domain-containing protein |
| FGRRES_09053 | 1.17 | 9.63E-08 | 5.46E-06 | hypothetical protein |
| FGRRES_03859 | 1.16 | 1.01E-05 | 3.56E-04 | hypothetical protein |
| FGRRES_10934 | 1.16 | 4.66E-06 | 1.79E-04 | allantoinase |
| FGRRES_09103 | 1.15 | 1.48E-04 | 3.73E-03 | 2og-fe oxygenase superfamily protein |
| FGRRES_03063 | 1.15 | 3.46E-10 | 3.30E-08 | ammonium permease mepa |
| FGRRES_00740 | 1.13 | 5.56E-08 | 3.32E-06 | urease |
| FGRRES_06551_M | 1.09 | 2.05E-04 | 4.95E-03 | gaba permease |
| FGRRES_02067_M | 1.09 | 1.85E-03 | 3.19E-02 | beta- insoluble isoenzyme cwinv1 |
| FGRRES_07980 | 1.07 | 2.15E-03 | 3.62E-02 | hypothetical protein |
| FGRRES_02927_M | 1.07 | 7.76E-04 | 1.55E-02 | aaa family atpase |
| FGRRES_08528 | 1.06 | 1.13E-04 | 2.98E-03 | phosphoadenosine phosphosulfate reductase |
| FGRRES_04667 | 1.05 | 1.17E-05 | 4.06E-04 | alpha-ketoglutarate-dependent sulfonate dioxygenase |
| FGRRES_16300_M | 1.05 | 1.99E-03 | 3.38E-02 | integral membrane protein |
| FGRRES_09599 | 1.04 | 7.58E-10 | 6.56E-08 | l-iditol 2-dehydrogenase |
| FGRRES_16109 | 1.03 | 2.90E-06 | 1.17E-04 | heterokaryon incompatibility protein |
| FGRRES_16058 | 1.01 | 1.43E-03 | 2.58E-02 | udp- transferase |
| FGRRES_10094 | -8.68 | 4.49E-245 | 5.09E-241 | polyamine transport protein |
| FGRRES_17549 | -3.62 | 1.78E-14 | 3.36E-12 | calcium-independent phospholipase a2 |
| FGRRES_11408 | -3.32 | 1.98E-25 | 1.87E-22 | ethyl tert-butyl ether degradation |
| FGRRES_03583 | -3.21 | 3.53E-09 | 2.74E-07 | triacylglycerol lipase v precursor |
| FGRRES_02296 | -3.08 | 1.15E-11 | 1.42E-09 | aldehyde dehydrogenase (nad+) |
| FGRRES_01756_M | -2.84 | 7.87E-19 | 2.88E-16 | hypothetical protein |
| FGRRES_02935 | -2.79 | 2.02E-04 | 4.89E-03 | hypothetical protein |
| FGRRES_10551 | -2.76 | 4.96E-56 | 2.81E-52 | killer toxin kp4 family protein |
| FGRRES_11132 | -2.72 | 2.81E-24 | 2.27E-21 | cyanamide hydratase |
| FGRRES_07743 | -2.60 | 1.94E-14 | 3.61E-12 | hypothetical protein |
| FGRRES_03925 | -2.59 | 5.85E-39 | 1.66E-35 | hypothetical protein |
| FGRRES_11174_M | -2.46 | 3.97E-32 | 6.43E-29 | calcium-independent phospholipase a2-gamma |
| FGRRES_15686 | -2.42 | 3.36E-07 | 1.71E-05 | tetracycline efflux protein |

| Gene ID | logFC* | P-Value | FDR[#] | blastp annotation |
|---|---|---|---|---|
| FGRRES_07386 | -2.39 | 9.13E-08 | 5.21E-06 | hypothetical protein |
| FGRRES_16263 | -2.32 | 3.44E-14 | 5.83E-12 | hypothetical protein |
| FGRRES_00050 | -2.31 | 3.11E-08 | 1.94E-06 | isoprenylcysteine alpha-carbonyl methylesterase icmel1 |
| FGRRES_07742 | -2.30 | 4.51E-09 | 3.43E-07 | serine threonine protein kinase |
| FGRRES_08076 | -2.24 | 2.60E-33 | 4.91E-30 | hypothetical protein |
| FGRRES_15875 | -2.22 | 1.28E-15 | 3.09E-13 | hypothetical protein |
| FGRRES_13962 | -2.21 | 1.10E-17 | 3.47E-15 | alcohol dehydrogenase |
| FGRRES_16870 | -2.19 | 1.64E-03 | 2.89E-02 | hypothetical protein |
| FGRRES_12586 | -2.18 | 1.70E-20 | 7.42E-18 | beta-glucosidase i |
| FGRRES_06087 | -2.16 | 1.23E-07 | 6.79E-06 | hypothetical protein |
| FGRRES_00136 | -2.11 | 4.43E-43 | 1.67E-39 | neutral amino acid permease |
| FGRRES_12177_M | -2.08 | 7.67E-06 | 2.76E-04 | hypothetical protein |
| FGRRES_10816 | -2.07 | 2.37E-09 | 1.92E-07 | mip family channel protein |
| FGRRES_07238 | -2.07 | 1.87E-04 | 4.56E-03 | beta- exoglucanase |
| FGRRES_17544 | -2.06 | 2.04E-11 | 2.29E-09 | phenazine biosynthesis protein |
| FGRRES_05839 | -2.06 | 2.86E-18 | 9.83E-16 | sugar transporter stl1 |
| FGRRES_03203 | -2.03 | 9.81E-05 | 2.64E-03 | hypothetical protein |
| FGRRES_20114 | -2.03 | 5.42E-09 | 4.07E-07 | hypothetical protein |
| FGRRES_00422 | -2.00 | 3.75E-04 | 8.31E-03 | hypothetical protein |
| FGRRES_01695_M | -1.97 | 1.09E-09 | 9.09E-08 | hypothetical protein |
| FGRRES_09621 | -1.97 | 6.51E-14 | 1.04E-11 | hypothetical protein |
| FGRRES_16093 | -1.97 | 1.32E-05 | 4.50E-04 | hypothetical protein |
| FGRRES_08122 | -1.96 | 3.41E-35 | 7.75E-32 | cell wall protein |
| FGRRES_12495 | -1.96 | 1.15E-19 | 4.67E-17 | trans-aconitate 2-methyltransferase |
| FGRRES_08830 | -1.95 | 2.76E-19 | 1.05E-16 | atpase |
| FGRRES_02216 | -1.92 | 7.65E-22 | 4.34E-19 | lipoxygenase 1 |
| FGRRES_13964 | -1.90 | 2.58E-24 | 2.25E-21 | c2h2 finger domain containing protein |
| FGRRES_04288 | -1.90 | 9.36E-24 | 6.64E-21 | 26s proteasome subunit rpn4 |
| FGRRES_17245 | -1.87 | 2.53E-08 | 1.64E-06 | hypothetical protein |
| FGRRES_00404 | -1.86 | 2.48E-15 | 5.41E-13 | acetate regulatory dna binding protein |
| FGRRES_11413 | -1.86 | 3.00E-12 | 4.01E-10 | acyl- transferase carnitine dehydratase |
| FGRRES_13963 | -1.86 | 9.86E-11 | 1.03E-08 | 3-hydroxybutyryl-dehydrogenase |
| FGRRES_01609 | -1.85 | 2.73E-04 | 6.27E-03 | hypothetical protein |
| FGRRES_00457 | -1.85 | 2.85E-26 | 3.23E-23 | 2-heptaprenyl- -naphthoquinone |

| Gene ID | logFC* | P-Value | FDR# | blastp annotation |
|---|---|---|---|---|
| FGRRES_17106 | -1.84 | 3.46E-12 | 4.46E-10 | peptidase m12 |
| FGRRES_02560 | -1.84 | 4.55E-24 | 3.44E-21 | secreted protein nis1 |
| FGRRES_02986 | -1.84 | 3.24E-18 | 1.08E-15 | hypothetical protein |
| FGRRES_09329 | -1.84 | 5.76E-17 | 1.72E-14 | atpase |
| FGRRES_13449 | -1.83 | 4.79E-09 | 3.62E-07 | hypothetical protein |
| FGRRES_12432 | -1.82 | 2.32E-04 | 5.50E-03 | hypothetical protein |
| FGRRES_03402 | -1.82 | 6.92E-08 | 4.05E-06 | acid phosphatase |
| FGRRES_16541 | -1.81 | 1.57E-19 | 6.14E-17 | hypothetical protein |
| FGRRES_02686 | -1.80 | 5.15E-04 | 1.09E-02 | ribonuclease trv |
| FGRRES_09825_M | -1.79 | 2.13E-20 | 8.96E-18 | hypothetical protein |
| FGRRES_03421 | -1.78 | 7.63E-04 | 1.54E-02 | hypothetical protein |
| FGRRES_11545 | -1.77 | 4.45E-08 | 2.73E-06 | hypothetical protein |
| FGRRES_12920 | -1.77 | 1.80E-03 | 3.14E-02 | stress responsive a b barrel domain-containing protein |
| FGRRES_03354 | -1.77 | 3.31E-09 | 2.59E-07 | involved in nonactin biosynthesis |
| FGRRES_07895 | -1.76 | 8.39E-26 | 8.66E-23 | hypothetical protein |
| FGRRES_10336 | -1.76 | 1.13E-03 | 2.12E-02 | methyltransferase type 11 protein |
| FGRRES_02279 | -1.76 | 9.34E-09 | 6.66E-07 | copper amine oxidase 1 |
| FGRRES_12240 | -1.75 | 7.25E-06 | 2.64E-04 | small s protein |
| FGRRES_08659 | -1.75 | 6.42E-22 | 3.98E-19 | hypothetical protein |
| FGRRES_12349 | -1.75 | 9.99E-21 | 4.53E-18 | hypothetical protein |
| FGRRES_02035 | -1.74 | 1.52E-09 | 1.25E-07 | vegetatible incompatibility protein het-e-1 |
| FGRRES_04458 | -1.74 | 1.23E-03 | 2.27E-02 | nitric oxide dioxygenase |
| FGRRES_16025 | -1.73 | 4.04E-30 | 5.73E-27 | hypothetical protein |
| FGRRES_04761_M | -1.71 | 6.27E-11 | 6.71E-09 | hypothetical protein |
| FGRRES_04626 | -1.70 | 5.36E-10 | 4.91E-08 | hypothetical protein |
| FGRRES_16442 | -1.69 | 1.89E-26 | 2.38E-23 | cytochrome-b5 reductase |
| FGRRES_03166_M | -1.69 | 2.21E-13 | 3.34E-11 | hypothetical protein |
| FGRRES_17466 | -1.68 | 1.43E-09 | 1.18E-07 | immune-responsive protein 1 |
| FGRRES_16465 | -1.67 | 1.70E-12 | 2.35E-10 | srp40-suppressor of mutant ac40 of rna polymerase i and iii |
| FGRRES_05008 | -1.67 | 2.19E-22 | 1.46E-19 | alpha beta hydrolase |
| FGRRES_10583 | -1.67 | 1.54E-11 | 1.78E-09 | hypothetical protein |
| FGRRES_11407 | -1.65 | 2.22E-06 | 9.15E-05 | aif1 apoptosis-inducing factor |
| FGRRES_11234_M | -1.65 | 3.49E-07 | 1.76E-05 | alpha- -mannosyltransferase |
| FGRRES_03502_M | -1.65 | 5.83E-16 | 1.54E-13 | hypothetical protein |
| FGRRES_03445 | -1.65 | 6.67E-22 | 3.98E-19 | hypothetical protein |

| Gene ID | logFC* | P-Value | FDR# | blastp annotation |
|---|---|---|---|---|
| FGRRES_11667_M | -1.65 | 3.49E-05 | 1.08E-03 | hypothetical protein |
| FGRRES_00814 | -1.64 | 8.24E-19 | 2.92E-16 | tim barrel metal-dependent hydrolase |
| FGRRES_09741 | -1.62 | 2.21E-21 | 1.19E-18 | hypothetical protein |
| FGRRES_07384 | -1.61 | 8.81E-21 | 4.17E-18 | hypothetical protein |
| FGRRES_11449 | -1.61 | 3.84E-05 | 1.17E-03 | hypothetical protein |
| FGRRES_11040 | -1.60 | 8.86E-04 | 1.73E-02 | glutathione s-transferase |
| FGRRES_01778 | -1.59 | 1.96E-11 | 2.25E-09 | hypothetical protein |
| FGRRES_07550 | -1.58 | 4.67E-04 | 1.00E-02 | multidrug resistance protein |
| FGRRES_04182 | -1.58 | 7.41E-21 | 3.66E-18 | carnitine transporter |
| FGRRES_11088 | -1.54 | 1.27E-04 | 3.31E-03 | na(+) h(+) antiporter |
| FGRRES_11391 | -1.54 | 1.56E-03 | 2.76E-02 | hypothetical protein |
| FGRRES_07425 | -1.52 | 3.48E-16 | 9.39E-14 | protein sfk1 |
| FGRRES_03347_M | -1.52 | 5.00E-21 | 2.58E-18 | pot family proton-dependent oligopeptide transporter |
| FGRRES_11175 | -1.51 | 2.95E-09 | 2.33E-07 | hypothetical protein |
| FGRRES_05848 | -1.50 | 3.67E-18 | 1.19E-15 | mfs sit siderophore-iron:h+ symporter |
| FGRRES_17404 | -1.50 | 8.66E-04 | 1.70E-02 | xyloglucan endo-transglycosylase-like protein |
| FGRRES_03330 | -1.49 | 1.25E-10 | 1.28E-08 | multidrug resistance protein |
| FGRRES_04371 | -1.49 | 1.17E-10 | 1.21E-08 | hypothetical protein |
| FGRRES_07597 | -1.47 | 6.68E-06 | 2.46E-04 | hypothetical protein |
| FGRRES_10617 | -1.47 | 3.73E-09 | 2.88E-07 | nonribosomal peptide synthetase |
| FGRRES_04698 | -1.47 | 1.90E-07 | 1.02E-05 | speckle-type poz protein |
| FGRRES_04732 | -1.47 | 1.50E-04 | 3.78E-03 | 6-hydroxy-d-nicotine oxidase |
| FGRRES_07739_M | -1.46 | 3.18E-07 | 1.63E-05 | hypothetical protein |
| FGRRES_02278 | -1.46 | 2.58E-03 | 4.24E-02 | amino acid permease |
| FGRRES_09972 | -1.46 | 6.53E-14 | 1.04E-11 | hypothetical protein |
| FGRRES_00765 | -1.46 | 1.70E-03 | 2.99E-02 | nitric oxide dioxygenase |
| FGRRES_10497_M | -1.45 | 1.50E-13 | 2.33E-11 | metallo-beta-lactamase family protein |
| FGRRES_01693 | -1.45 | 2.52E-14 | 4.43E-12 | hypothetical protein |
| FGRRES_02273 | -1.45 | 3.78E-07 | 1.87E-05 | aldehyde dehydrogenase (nad+) |
| FGRRES_03285 | -1.44 | 1.35E-06 | 5.81E-05 | short-chain dehydrogenase reductase sdr |
| FGRRES_07557 | -1.44 | 3.87E-06 | 1.54E-04 | transcription co-repressor gal80 |
| FGRRES_09078 | -1.43 | 2.47E-07 | 1.30E-05 | neutral amino acid permease |
| FGRRES_03355 | -1.43 | 3.37E-11 | 3.67E-09 | nadh:flavin oxidoreductase nadh oxidase |
| FGRRES_11044 | -1.43 | 4.56E-13 | 6.81E-11 | protein kes1 |

| Gene ID | logFC* | P-Value | FDR# | blastp annotation |
|---|---|---|---|---|
| FGRRES_09142 | -1.43 | 1.64E-14 | 3.15E-12 | kelch domain-containing protein 8a |
| FGRRES_10773 | -1.42 | 4.37E-05 | 1.29E-03 | isoflavone reductase p3 |
| FGRRES_00878 | -1.41 | 3.22E-05 | 1.00E-03 | hypothetical protein |
| FGRRES_16267 | -1.41 | 1.71E-04 | 4.25E-03 | stage v sporulation protein k |
| FGRRES_02262 | -1.41 | 2.18E-13 | 3.34E-11 | glycoside hydrolase family 16 |
| FGRRES_17681_M | -1.40 | 2.44E-14 | 4.40E-12 | caspase domain-containing protein |
| FGRRES_15874 | -1.39 | 4.34E-04 | 9.38E-03 | hypothetical protein |
| FGRRES_10383 | -1.38 | 1.40E-04 | 3.61E-03 | hypothetical protein |
| FGRRES_03761 | -1.38 | 8.02E-05 | 2.22E-03 | n1-acetylpolyamine oxidase |
| FGRRES_05928 | -1.38 | 1.22E-05 | 4.22E-04 | hypothetical protein |
| FGRRES_04656 | -1.37 | 7.03E-10 | 6.20E-08 | beta-lactamase-like 1 |
| FGRRES_11936 | -1.37 | 3.10E-16 | 8.59E-14 | hypothetical protein |
| FGRRES_04887 | -1.36 | 3.33E-12 | 4.34E-10 | cg121_gibze ame: full=ekc keops complex subunit cgi121 |
| FGRRES_02138 | -1.35 | 1.49E-07 | 8.11E-06 | cytochrome p450 oxidoreductase |
| FGRRES_10513_M | -1.35 | 1.50E-15 | 3.48E-13 | litaf-like zinc finger domain-containing protein |
| FGRRES_16181 | -1.35 | 6.29E-05 | 1.77E-03 | metallo-beta-lactamase family protein |
| FGRRES_17220 | -1.35 | 9.03E-09 | 6.52E-07 | hypothetical protein |
| FGRRES_05375 | -1.34 | 1.31E-17 | 4.03E-15 | betaine-aldehyde dehydrogenase |
| FGRRES_04616 | -1.34 | 2.05E-03 | 3.48E-02 | glutathione-dependent formaldehyde-activating gfa protein |
| FGRRES_10224 | -1.34 | 8.62E-05 | 2.36E-03 | hypothetical protein |
| FGRRES_16227 | -1.34 | 9.41E-10 | 8.03E-08 | peroxisomal short-chain alcohol dehydrogenase |
| FGRRES_01763 | -1.33 | 2.12E-04 | 5.09E-03 | hydrophobin 1 |
| FGRRES_04012 | -1.33 | 1.17E-05 | 4.06E-04 | oxidoreductase |
| FGRRES_17101_M | -1.33 | 1.06E-07 | 5.94E-06 | ankyrin repeat protein |
| FGRRES_09287 | -1.32 | 3.39E-14 | 5.82E-12 | sexual differentiation process protein isp4 |
| FGRRES_16217 | -1.32 | 2.55E-08 | 1.64E-06 | -like family domain-containing protein 1 |
| FGRRES_11979 | -1.31 | 8.80E-12 | 1.10E-09 | protein-tyrosine phosphatase |
| FGRRES_10790 | -1.31 | 6.01E-13 | 8.85E-11 | acyl- dehydrogenase |
| FGRRES_06719 | -1.30 | 4.37E-11 | 4.72E-09 | hypothetical protein |
| FGRRES_01330 | -1.30 | 8.19E-15 | 1.66E-12 | thioesterase family protein |
| FGRRES_05589 | -1.30 | 5.26E-10 | 4.85E-08 | atp-binding cassette protein |
| FGRRES_07674 | -1.29 | 9.35E-07 | 4.21E-05 | hypothetical protein |
| FGRRES_10507 | -1.29 | 2.06E-14 | 3.77E-12 | transmembrane protein 53-a |

| Gene ID | logFC* | P-Value | FDR# | blastp annotation |
|---|---|---|---|---|
| FGRRES_02886 | -1.29 | 1.04E-03 | 2.01E-02 | isoflavone reductase family protein |
| FGRRES_09267 | -1.29 | 1.28E-11 | 1.55E-09 | mitochondrial chaperone bcs1 |
| FGRRES_03678 | -1.28 | 4.89E-07 | 2.35E-05 | hypothetical protein |
| FGRRES_09211 | -1.28 | 1.34E-03 | 2.43E-02 | hypothetical protein |
| FGRRES_05374 | -1.28 | 2.30E-15 | 5.11E-13 | choline dehydrogenase |
| FGRRES_02213 | -1.28 | 1.22E-04 | 3.20E-03 | hypothetical protein |
| FGRRES_03565 | -1.28 | 1.48E-16 | 4.30E-14 | nitrogen starvation-induced glutamine rich protein |
| FGRRES_01513 | -1.28 | 1.36E-13 | 2.14E-11 | hypothetical protein |
| FGRRES_04760_M | -1.27 | 1.03E-09 | 8.70E-08 | hypothetical protein |
| FGRRES_00150 | -1.27 | 1.38E-15 | 3.27E-13 | zinc-binding dehydrogenase |
| FGRRES_07299 | -1.27 | 2.54E-14 | 4.43E-12 | transferase mitochondrial |
| FGRRES_11343 | -1.27 | 2.93E-03 | 4.70E-02 | integral membrane protein |
| FGRRES_01587 | -1.25 | 7.81E-12 | 9.85E-10 | ankyrin repeat domain-containing protein 23 |
| FGRRES_01972 | -1.24 | 5.55E-15 | 1.15E-12 | bifunctional p-450:nadph-p450 reductase |
| FGRRES_08123 | -1.24 | 5.86E-08 | 3.46E-06 | hypothetical protein |
| FGRRES_01331 | -1.24 | 3.58E-14 | 5.97E-12 | hypothetical protein |
| FGRRES_07713_M | -1.23 | 1.82E-05 | 5.94E-04 | hypothetical protein |
| FGRRES_03185 | -1.23 | 4.05E-05 | 1.23E-03 | hypothetical protein |
| FGRRES_08979 | -1.23 | 1.76E-15 | 4.00E-13 | family protein |
| FGRRES_02942 | -1.23 | 1.49E-03 | 2.66E-02 | hypothetical protein |
| FGRRES_12066 | -1.23 | 2.55E-08 | 1.64E-06 | dsba oxidoreductase |
| FGRRES_16798 | -1.23 | 7.12E-13 | 1.04E-10 | nadh dehydrogenase |
| FGRRES_03674_M | -1.23 | 8.18E-07 | 3.73E-05 | filamentous hemagglutinin adhesin |
| FGRRES_15009 | -1.22 | 7.28E-06 | 2.64E-04 | conidiation protein con-6 |
| FGRRES_06130 | -1.22 | 1.07E-15 | 2.76E-13 | hypothetical protein |
| FGRRES_01736_M | -1.22 | 1.20E-14 | 2.39E-12 | hypothetical protein |
| FGRRES_05937 | -1.22 | 2.64E-15 | 5.64E-13 | phospholipase a2 |
| FGRRES_00817 | -1.22 | 4.21E-14 | 6.93E-12 | leucine rich repeat domain protein |
| FGRRES_11104 | -1.22 | 2.95E-07 | 1.54E-05 | triacylglycerol lipase ii precursor |
| FGRRES_02137 | -1.22 | 7.24E-07 | 3.37E-05 | cytochrome-b5 reductase |
| FGRRES_04213 | -1.21 | 1.36E-14 | 2.67E-12 | hypothetical protein |
| FGRRES_04207 | -1.21 | 1.10E-15 | 2.78E-13 | glutamine rich nitrogen starvation-induced |
| FGRRES_10920 | -1.21 | 2.39E-07 | 1.26E-05 | endo-polygalacturonase 6 |
| FGRRES_04861 | -1.20 | 1.13E-04 | 2.98E-03 | nad-dependent epimerase dehydratase |

| Gene ID | logFC* | P-Value | FDR# | blastp annotation |
|---|---|---|---|---|
| FGRRES_02018 | -1.20 | 1.76E-12 | 2.41E-10 | duf500 domain protein |
| FGRRES_17457 | -1.19 | 2.15E-03 | 3.61E-02 | 3-oxoacyl-[acyl-carrier protein] reductase |
| FGRRES_07488 | -1.19 | 4.06E-15 | 8.53E-13 | hypothetical protein |
| FGRRES_04917 | -1.19 | 6.88E-06 | 2.52E-04 | hypothetical protein |
| FGRRES_10569 | -1.18 | 4.64E-07 | 2.25E-05 | zinc finger protein |
| FGRRES_15022 | -1.18 | 2.25E-04 | 5.36E-03 | hypothetical protein |
| FGRRES_07582 | -1.17 | 7.05E-10 | 6.20E-08 | mfs sp sugar:h+ symporter |
| FGRRES_03725 | -1.17 | 7.52E-13 | 1.08E-10 | mfs-multidrug-resistance transporter |
| FGRRES_10969 | -1.16 | 1.25E-12 | 1.77E-10 | hypothetical protein |
| FGRRES_00176 | -1.16 | 6.55E-11 | 6.95E-09 | isocitrate lyase |
| FGRRES_01239 | -1.15 | 3.84E-10 | 3.60E-08 | hypothetical protein |
| FGRRES_13169 | -1.14 | 4.53E-05 | 1.33E-03 | glycosyl transferase family protein |
| FGRRES_07390 | -1.14 | 1.49E-10 | 1.50E-08 | hypothetical protein |
| FGRRES_04114 | -1.13 | 1.68E-06 | 7.03E-05 | adenosine deaminase |
| FGRRES_06735_M | -1.13 | 2.29E-10 | 2.24E-08 | inositol-pentakisphosphate 2-kinase |
| FGRRES_08238 | -1.13 | 3.76E-04 | 8.33E-03 | hypothetical protein |
| FGRRES_05296 | -1.13 | 1.29E-11 | 1.55E-09 | hypothetical protein |
| FGRRES_09081 | -1.13 | 1.14E-06 | 5.02E-05 | mfs sp general alpha glucoside:h+ symporter |
| FGRRES_10581 | -1.12 | 7.26E-09 | 5.31E-07 | ankyrin repeat protein |
| FGRRES_01734_M | -1.12 | 4.85E-08 | 2.94E-06 | quinate transport protein |
| FGRRES_10212 | -1.12 | 2.75E-11 | 3.03E-09 | rot1 precursor |
| FGRRES_11200 | -1.12 | 2.82E-09 | 2.23E-07 | heterokaryon incompatibility protein |
| FGRRES_07908 | -1.12 | 1.40E-08 | 9.70E-07 | glycerol dehydrogenase protein |
| FGRRES_07907 | -1.11 | 5.46E-09 | 4.07E-07 | phosphatidyl synthase protein |
| FGRRES_08114_M | -1.11 | 8.24E-04 | 1.64E-02 | 5-methylthioadenosine s-adenosylhomocysteine deaminase |
| FGRRES_06917 | -1.10 | 3.30E-07 | 1.69E-05 | hypothetical protein |
| FGRRES_03586 | -1.10 | 1.88E-08 | 1.27E-06 | hypothetical protein |
| FGRRES_04071_M | -1.10 | 7.81E-11 | 8.20E-09 | pua rna binding domain-containing protein |
| FGRRES_03363 | -1.10 | 7.67E-07 | 3.55E-05 | long-chain-fatty-acid-- ligase |
| FGRRES_03448 | -1.10 | 6.07E-08 | 3.57E-06 | altered inheritance rate of mitochondria protein 38 like protein |
| FGRRES_02217 | -1.10 | 3.77E-04 | 8.33E-03 | catalase |
| FGRRES_08681 | -1.09 | 9.80E-05 | 2.64E-03 | hypothetical protein |

| Gene ID | logFC* | P-Value | FDR[#] | blastp annotation |
|---|---|---|---|---|
| FGRRES_08252 | -1.09 | 1.49E-04 | 3.74E-03 | endoglucanase type c |
| FGRRES_08291 | -1.09 | 1.39E-11 | 1.64E-09 | hypothetical protein |
| FGRRES_01686 | -1.09 | 2.22E-11 | 2.47E-09 | alcohol dehydrogenase (nadp+) |
| FGRRES_06508 | -1.08 | 1.23E-03 | 2.27E-02 | aat family amino acid transporter |
| FGRRES_10506 | -1.08 | 7.77E-07 | 3.58E-05 | transporter mch4 |
| FGRRES_06460_M | -1.08 | 1.48E-04 | 3.74E-03 | hypothetical protein |
| FGRRES_09563 | -1.08 | 1.62E-12 | 2.26E-10 | ring-like domain-containing protein |
| FGRRES_01606 | -1.08 | 9.43E-09 | 6.69E-07 | ca2+:h+ antiporter |
| FGRRES_11858 | -1.07 | 1.23E-05 | 4.22E-04 | hypothetical protein |
| FGRRES_02467 | -1.07 | 1.11E-03 | 2.10E-02 | isopenicillin n epimerase |
| FGRRES_17449 | -1.07 | 5.35E-08 | 3.21E-06 | disintegrin and metalloproteinase domain-containing protein b |
| FGRRES_03249 | -1.07 | 5.76E-12 | 7.34E-10 | glycerol-3-phosphate dehydrogenase |
| FGRRES_00453 | -1.07 | 3.92E-05 | 1.19E-03 | hypothetical protein |
| FGRRES_02221 | -1.07 | 5.36E-06 | 2.02E-04 | hypothetical protein |
| FGRRES_12386 | -1.07 | 2.38E-03 | 3.93E-02 | alcohol dehydrogenase 1 |
| FGRRES_12999 | -1.06 | 1.49E-05 | 4.96E-04 | hypothetical protein |
| FGRRES_02284 | -1.06 | 1.39E-11 | 1.64E-09 | gnat family |
| FGRRES_07564 | -1.06 | 9.41E-07 | 4.22E-05 | multidrug resistant protein |
| FGRRES_12021 | -1.06 | 1.02E-06 | 4.51E-05 | hypothetical protein |
| FGRRES_16768_M | -1.06 | 2.34E-10 | 2.25E-08 | zinc finger protein |
| FGRRES_10020 | -1.06 | 4.11E-08 | 2.55E-06 | triacylglycerol lipase |
| FGRRES_10474 | -1.05 | 2.23E-03 | 3.71E-02 | plastidic glucose transporter 4 |
| FGRRES_08073_M | -1.05 | 2.91E-08 | 1.84E-06 | hypothetical protein |
| FGRRES_16825 | -1.04 | 4.26E-10 | 3.96E-08 | atpase |
| FGRRES_08585 | -1.04 | 9.55E-05 | 2.59E-03 | palmitoyl- hydrolase |
| FGRRES_03370 | -1.04 | 1.52E-05 | 5.03E-04 | salicylate 1-monooxygenase |
| FGRRES_13442 | -1.04 | 1.25E-08 | 8.78E-07 | hypothetical protein |
| FGRRES_09235 | -1.04 | 6.69E-10 | 5.98E-08 | hypothetical protein |
| FGRRES_00296 | -1.03 | 3.10E-12 | 4.09E-10 | threonine ammonia- biosynthetic |
| FGRRES_07078 | -1.03 | 1.33E-10 | 1.35E-08 | c3hc4 zinc-binding integral peroxisomal membrane protein |
| FGRRES_16684 | -1.03 | 6.24E-07 | 2.92E-05 | hypothetical protein |
| FGRRES_02322 | -1.03 | 9.13E-04 | 1.78E-02 | major facilitator superfamily transporter |
| FGRRES_02061 | -1.03 | 8.63E-10 | 7.42E-08 | hypothetical protein |
| FGRRES_00698 | -1.03 | 6.19E-10 | 5.58E-08 | hypothetical protein |
| FGRRES_09757 | -1.03 | 8.52E-05 | 2.34E-03 | acyl-coenzyme a thioesterase 13 |

| Gene ID | logFC* | P-Value | FDR[#] | blastp annotation |
|---|---|---|---|---|
| FGRRES_15916 | -1.03 | 1.40E-08 | 9.70E-07 | aspartyl-trna synthetase |
| FGRRES_08690 | -1.02 | 1.76E-10 | 1.74E-08 | zsp1 zinc sensitive phenotype 1 |
| FGRRES_03292 | -1.02 | 1.41E-04 | 3.61E-03 | fungal specific transcription factor domain-containing protein |
| FGRRES_01159 | -1.02 | 3.93E-07 | 1.94E-05 | hypothetical protein |
| FGRRES_04240 | -1.02 | 5.72E-05 | 1.63E-03 | sodium- and chloride-dependent gaba transporter 1 |
| FGRRES_12331 | -1.02 | 1.41E-11 | 1.65E-09 | zinc-binding oxidoreductase |
| FGRRES_08657 | -1.01 | 7.19E-10 | 6.28E-08 | hypothetical protein |
| FGRRES_00080 | -1.01 | 1.36E-04 | 3.52E-03 | arylamine n-acetyltransferase |
| FGRRES_04560 | -1.01 | 1.41E-06 | 6.07E-05 | hypothetical protein |
| FGRRES_08562 | -1.01 | 2.32E-10 | 2.25E-08 | hypothetical protein |
| FGRRES_03081 | -1.01 | 2.13E-04 | 5.10E-03 | hypothetical protein |
| FGRRES_17360 | -1.01 | 1.55E-04 | 3.89E-03 | hypothetical protein |
| FGRRES_12850 | -1.01 | 5.95E-04 | 1.24E-02 | hypothetical protein |

*FC, fold change

[#]FDR, false discovery rate

**Table. S3 Primer sequences used for vector construction**

| Primer | Sequence (5'→3')[ab] |
|---|---|
| 094N-F | <u>CGACTCACTATAGGGCGAATTGGGTACTCAAATTGG</u>GTGTGCTGGGCGTAAGAAAGG |
| 094R-F | <u>CAGATCTTGGCTTTCGTAGGAACCCAATCTTCA</u>ATGACCGATGCTCGTTTGCCTC |
| 094-R | <u>CACCACCCCGGTGAACAGCTCCTCGCCCTTGCTCAC</u>AACATCCTTGCGAGAGTTGCTTG |
| 094E-F | AAGCAATGTCAACTCCGACAAGGACT**G**GAAGTCTGGCACTCTTCAGGCGAAAG |
| 094E-R | CTTTCGCCTGAAGAGTGCCAGACTTC**C**AGTCCTTGTCGGAGTTGACATTGCTT |
| 094S-F | AAGCAATGTCAACTCCGACAAGGACTA**A**AAGTCTGGCACTCTTCAGGCGAAAG |
| 094S-R | CTTTCGCCTGAAGAGTGCCAGACTT**T**TAGTCCTTGTCGGAGTTGACATTGCTT |

a. Underlined sequences represent homologous sequences required for recombination.

b. Bases in bold and italics represent mutation sites
